# Interleukin‐6 neutralization ameliorates symptoms in prematurely aged mice

**DOI:** 10.1111/acel.13285

**Published:** 2021-01-03

**Authors:** Stefano Squarzoni, Elisa Schena, Patrizia Sabatelli, Elisabetta Mattioli, Cristina Capanni, Vittoria Cenni, Maria Rosaria D'Apice, Davide Andrenacci, Giuseppe Sarli, Valeria Pellegrino, Anna Festa, Fabio Baruffaldi, Gianluca Storci, Massimiliano Bonafè, Catia Barboni, Mara Sanapo, Anna Zaghini, Giovanna Lattanzi

**Affiliations:** ^1^ CNR Institute of Molecular Genetics “Luigi Luca Cavalli‐Sforza” Unit of Bologna Bologna Italy; ^2^ IRCCS Istituto Ortopedico Rizzoli Bologna Italy; ^3^ Tor Vergata Hospital Medical Genetics Laboratory Rome Italy; ^4^ Department of Veterinary Medical Sciences University of Bologna Bologna Italy; ^5^ Laboratory of Medical Technology IRCCS Istituto Ortopedico Rizzoli Bologna Italy; ^6^ Department of Experimental, Diagnostic and Specialty Medicine University of Bologna Bologna Italy

**Keywords:** accelerated aging, ageing, anti‐aging, cellular senescence, cytokines, inflammation, laminopathies, nuclear lamina

## Abstract

Hutchinson–Gilford progeria syndrome (HGPS) causes premature aging in children, with adipose tissue, skin and bone deterioration, and cardiovascular impairment. In HGPS cells and mouse models, high levels of interleukin‐6, an inflammatory cytokine linked to aging processes, have been detected. Here, we show that inhibition of interleukin‐6 activity by tocilizumab, a neutralizing antibody raised against interleukin‐6 receptors, counteracts progeroid features in both HGPS fibroblasts and *Lmna^G609G^* ^/^ *^G609G^* progeroid mice. Tocilizumab treatment limits the accumulation of progerin, the toxic protein produced in HGPS cells, rescues nuclear envelope and chromatin abnormalities, and attenuates the hyperactivated DNA damage response. In vivo administration of tocilizumab reduces aortic lesions and adipose tissue dystrophy, delays the onset of lipodystrophy and kyphosis, avoids motor impairment, and preserves a good quality of life in progeroid mice. This work identifies tocilizumab as a valuable tool in HGPS therapy and, speculatively, in the treatment of a variety of aging‐related disorders.

## Graphical abstract

Signs of premature ageing are improved by tocilizumab treatment. A study in a murine model of Hutchinson‐Gilford Progeria shows that neutralization of interleukin 6 preserves motor activity and slows‐down tissue deterioration.

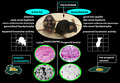

## 1 INTRODUCTION

HGPS is a rare disease that causes accelerated aging in children. Disease symptoms appear soon after birth and include skin abnormalities, alopecia, osteoporosis, and osteolysis with bone resorption at clavicles, phalanges and mandible, generalized lipodystrophy, and cardiovascular disorders leading to early death (Filgueiras‐Rama et al., 2018; Gonzalo et al., 2017; Hamczyk et al., 2018). Searching therapeutic strategies for HGPS is still a challenge. Although an ongoing clinical trial exploiting a farnesyltransferase inhibitor elicited several positive results (Gordon et al., 2018), significant increase in life span and slowdown of organismal aging were not achieved. Moreover, poor quality of life characterizes HGPS due to very early onset and progressive worsening of osteoporosis, lipodystrophy, articular impairment, and cardiovascular disorders, which are barely improved by current treatments (Gordon et al., 2018).

The molecular defect causing HGPS is an heterozygous mutation in the *LMNA* gene, which encodes five A type lamins by alternative splicing, including lamins A and C (Eriksson et al., 2003). Most HGPS cases are linked to the c.1824C>T;p.G608G silent mutation in *LMNA* gene, which activates an aberrant splicing (De Sandre‐Giovannoli et al., 2003; Eriksson et al., 2003). The aberrantly spliced gene product is translated as progerin, a truncated prelamin A form that undergoes farnesylation at its C‐terminus as wild‐type prelamin A, but cannot be fully processed. Thus, progerin is maintained in HGPS cells as a permanently farnesylated protein precursor (Eriksson et al., 2003) and causes chromatin disorganization, aberrant nuclear lamina interaction with transcription factors and chromatin‐binding proteins, upregulation of p21 and geroconversion of cells (Kreienkamp et al., 2016, 2018; Mattioli et al., 2018). Further, altered nucleo‐cytoskeleton interplay involving tubulins and defective ion channel expression or activity have been shown in progeroid cells (Larrieu et al., 2018; Zironi et al., 2018). The outcome of such an altered scenario seems to be an unscheduled activation of stress response, marked by persistence of DNA damage markers as phosphorylated histone H2AX (γ‐H2AX) and small telomeric DNA damage response RNAs (Aguado et al., 2019).

Progerin effects on the secretome have been observed in several preclinical models of HGPS (Gonzalo & Coll‐Bonfill, 2019; Kreienkamp et al., 2018; Osmanagic‐Myers et al., 2019; Osorio et al., 2012). In mice, it has been demonstrated that selective expression of progerin in endothelial cells causes dysregulation of circulating molecules and a condition leading to paracrine and profibrotic effects (Osmanagic‐Myers et al., 2019; Sun et al., 2020). Activation of the senescence‐associated secretory phenotype (SASP) by endothelium‐targeted progerin affected most mouse tissues and induced premature aging in the whole organism (Sun et al., 2020). Systemic effects linked to aberrant NF‐kB signaling and interleukin 6 (IL6) increase have been observed in *Lmna^G609G^* ^/^ *^G609G^* and *Zmpste24* ^−/−^ progeroid mice featuring progerin or prelamin A accumulation, while anti‐inflammatory drugs have been shown to extend life span (Osorio et al., 2011, 2012). Moreover, recent studies showed that aberrant activation of JAK‐STAT signaling occurs in HGPS cells and animal models and triggers SASP with increase of IL6 and IL8 (Griveau et al., 2020; Liu et al., 2019). Intriguingly, prelamin A‐dependent SASP activation, including IL6 hypersecretion, has been also observed in human vascular smooth muscle cells undergoing calcification (Liu et al., 2013), a condition that is found in HGPS and contributes to disease severity (Gordon et al., 2016).

IL6 behaves as a pro‐inflammatory cytokine, with features of anti‐inflammatory molecule under certain conditions. In particular, canonical IL6 signaling, which relies on membrane‐bound receptor alpha (IL6Ra) and on the ubiquitous receptor GP130 (Baran et al., 2018), triggers anti‐inflammatory and pro‐regenerative pathways, and it is restricted to cells harboring the receptor on their membrane, mainly hepatocytes and macrophages (Baran et al., 2018). Conversely, through the soluble IL6 receptor (sIL6R), IL6 stimulates pro‐inflammatory response and pro‐fibrotic processes in several cell types and target tissues (Baran et al., 2018). In this context, IL6 propagates inflammation signaling from cells that produce the cytokine to neighboring cells causing DNA damage in a sort of self‐fueling circle activated by various stress conditions (Fang et al., 2014; Rodier et al., 2009; Storci et al., 2019),

In the clinical practice, an anti‐IL6 receptor antibody (tocilizumab) is widely used to treat the abnormal inflammatory response that occurs in autoimmune diseases as rheumatoid arthritis and it is well tolerated by patients, even at very young age (Emery et al., 2019; Mallalieu et al., 2019; Mihara et al., 2011). The positive effect of the antibody relies on neutralization of IL6 activity through competition with soluble and membrane‐bound IL6 receptors (Mihara et al., 2011). Efficacy of antibody treatment has been demonstrated particularly in the osteoarticular system in both murine experimental models (Kamiya et al., 2019) and patients (Safy‐Khan et al., 2020). We reasoned that tocilizumab treatment could be beneficial in HGPS by reducing IL6‐related progeroid features and tested this hypothesis in cultured HGPS fibroblasts, *Lmna^G609G^* ^/^ *^G609G^* mouse cells, and in vivo in *Lmna^G609G^* ^/^ *^G609G^* mice (Osorio et al., 2011, 2012; Zaghini et al., 2020). We show here that tocilizumab counteracts aberrant differentiation of adipocytes and osteoblasts from *Lmna^G609G^* ^/^ *^G609G^* progeroid mice, improves adipose tissue dystrophy, aorta histological lesions, and skeletal deterioration, and positively impacts the overall condition of progeroid mice, while unexpectedly reducing progerin accumulation and its deleterious effects in mouse and human progeria cells.

## 2 RESULTS

### 2.1 IL6 secretion is increased in HGPS cells

**FIGURE 1:**
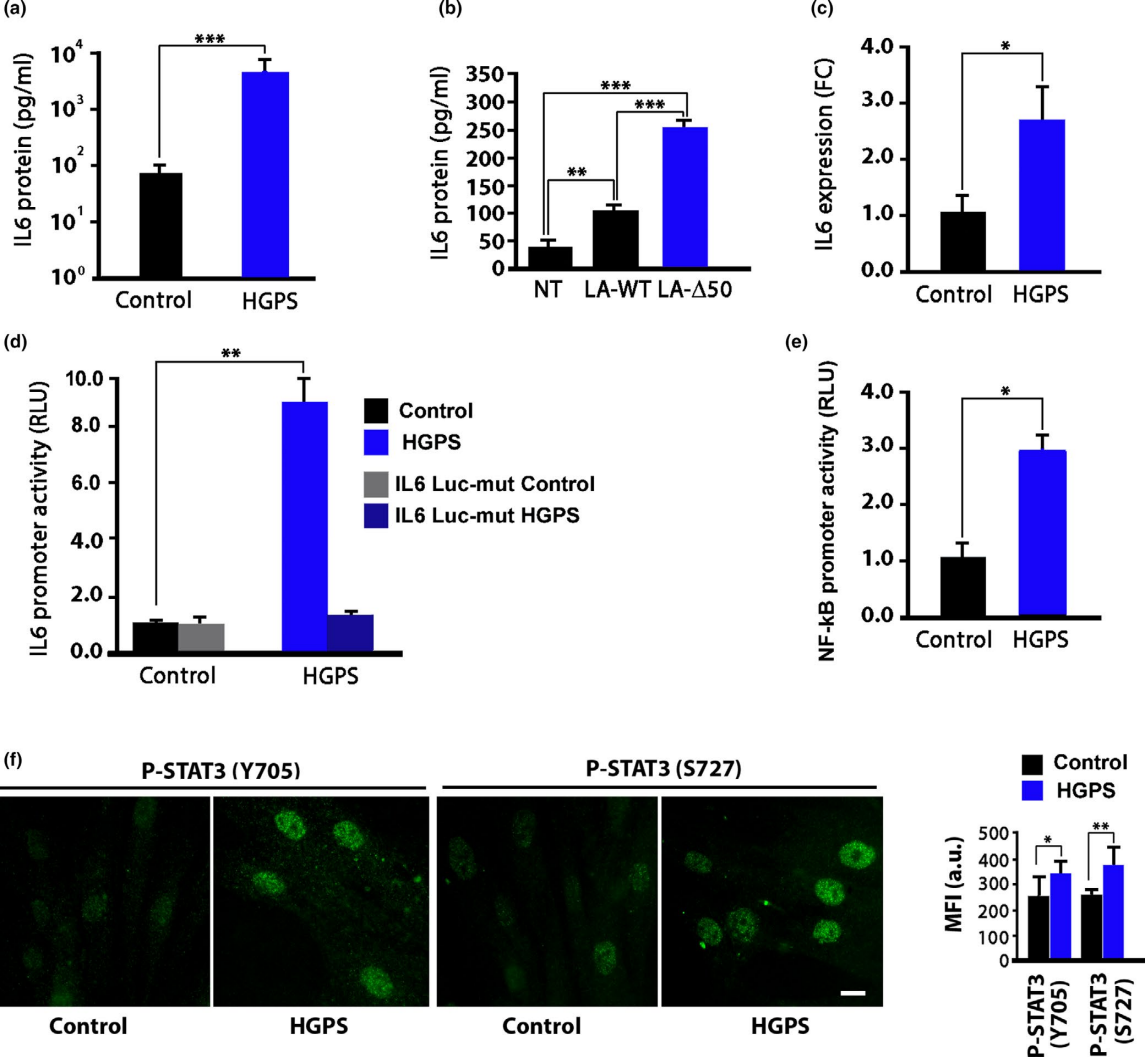
IL6 levels and activity are increased in HGPS cells. (a) IL6 secretion in culture media of normal human dermal fibroblasts (Control) or HGPS fibroblasts (HGPS) after 48 h in cell culture. IL6 levels were measured by ELISA. (b) IL6 secretion in culture media of HEK293 non‐transfected (NT) or transiently transfected with plasmids carrying WT‐*LMNA* (LA‐WT) or Δ50‐*LMNA*(LA‐Δ50) and kept in culture for 48 h after transfection. IL6 levels were measured by ELISA. (c) qRT‐PCR analysis of IL6 expression in control (Control) and HGPS fibroblasts (HGPS). (d) Activity of IL6 promoter in control (Control) and HGPS fibroblasts (HGPS) measured by a luciferase reporter assay. An IL6 luciferase mutant (IL6 Luc‐mut) was also expressed in normal and HGPS fibroblasts as a negative control. (e) Activity of NF‐kB promoter in control (Control) and HGPS fibroblasts (HGPS) measured by a luciferase reporter assay. (f) Immunofluorescence labeling of phosphorylated STAT3 in control (Control) and HGPS fibroblasts (HGPS). Mean fluorescence intensity values (MFI) are reported in the graph as arbitrary units (a.u.). Scale bar, 10 µm Three biological replicates were used in all analyses (panels a, b, d, e, f), except in qRT‐PCR (panel c, six biological replicates). Data are reported as means ± *SEM*. Statistically significant differences are indicated (*p < 0.05, **p < 0.01, ***p < 0.001)

Consistent with previous studies (Bidault et al., 2020; Liu et al., 2019), we observed that IL6 secretion is increased in HGPS fibroblasts carrying the classical G608G *LMNA* mutation and also in fibroblasts from Mandibuloacral Dysplasia, another *LMNA*‐linked progeroid syndrome (Cenni et al., 2018; Filesi et al., 2005) (Figure 1a and Figure S1a). Further, the *LMNA* delta 50 mutation causing progerin expression, when transiently expressed in human HEK293 cells, induced hypersecretion of IL6 (Figure 1b). Although overexpression of wild‐type *LMNA* elicited some increase in IL6 levels, much more significant enhancement of IL6 secretion was induced by progerin (Figure 1b) as well as by Mandibuloacral Dysplasia‐linked R527H‐mutated lamin A (Figure S1B). These results suggested that lamin A molecular defect was the primary cause of IL6 upregulation, which was linked to overexpression of IL6 gene (Figure 1c) and activation of IL6 promoter (Figure 1d). To confirm the latter finding, we used an IL6 luciferase mutant, which did not elicit any promoter activity signal neither in controls nor in HGPS fibroblasts (Figure 1D). Moreover, NF‐kB promoter was hyperactivated in HGPS cells (Figure 1e). We further observed that activated STAT3, a main effector of IL6 signaling, was accumulated to a significantly higher extent in the nucleus of HGPS fibroblasts relative to control fibroblast nuclei (Figure 1f). In fact, both Tyrosine 705 and Serine 727 STAT3 phosphorylation were increased in HGPS nuclei (Figure 1f).

### 2.2 Tocilizumab counteracts IL6 activity and bystander effects and progerin accumulation

**FIGURE 2:**
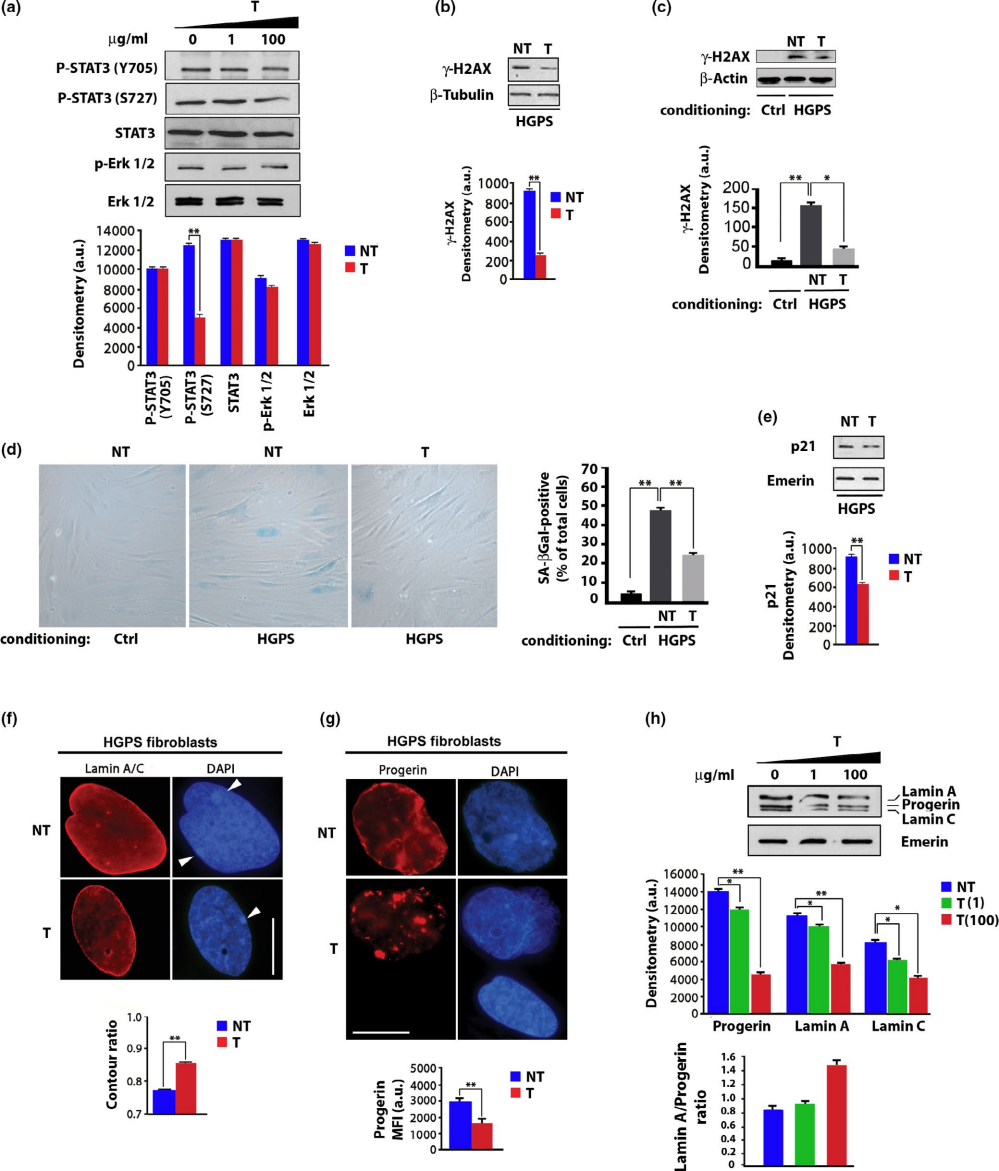
Tocilizumab reduces IL‐6 dependent STAT3 and γ‐H2AX activation and counteracts cellular senescence. (a) Western blot analysis of STAT3 phosphorylated on Tyrosine 705 (P‐STAT3 Y705) or Serine 727 (P‐STAT3 S727), STAT3, in untreated or tocilizumab‐treated HGPS fibroblasts (T). Tocilizumab dosage (µg/ml culture medium) is indicated in the upper row. In the graphs and all the following panels: NT, 0 µg/ml tocilizumab; T, 100 µg/ml tocilizumab. (b)Western blot analysis of γ‐H2AX in HGPS fibroblasts and (c) in normal human dermal fibroblasts subjected to conditioning with control fibroblasts (ctrl) or HGPS fibroblast medium (HGPS). β‐tubulin and β‐actin bands are shown as protein‐loading controls.(d) Senescence‐associated beta‐Galactosidase staining (SA‐βGal) of normal human dermal fibroblasts co‐cultured with control (ctrl) or HGPS fibroblasts (HGPS)left untreated (NT) or treated with tocilizumab (T).The percentage of SA‐βGal‐positive cells is reported in the graph. (e) Western blot analysis of p21 in HGPS fibroblasts. (f) Immunofluorescence analysis of Lamin A/C in HGPS fibroblasts untreated (NT) and treated (T) with tocilizumab. DAPI staining of DNA (blue) allows detection of facultative heterochromatin areas (arrowheads), which correspond to inactive X chromosome and appear duplicated in the untreated HGPS fibroblasts, but not in tocilizumab‐treated cells. Scale bar, 10 µm. Contour ratio of nuclei determined in HGPS fibroblasts left untreated (NT) or treated (T) with tocilizumab is reported in the graph. (g) Immunofluorescence analysis of progerin in HGPS fibroblasts untreated (NT) or treated with tocilizumab (T). Progerin MFI in HGPS nuclei is reported in the graph in arbitrary units (a.u.). (h) Western blot analysis of Progerin and Lamin A/C in HGPS fibroblasts left untreated (NT) or treated (T) with tocilizumab. Emerin bands are shown as protein‐loading controls. Densitometry of immunoblotted protein bands is reported in the upper graph. Lamin A to progerin ratio (calculated on mean densitometric values of each sample) is shown in the lower graph. Three biological replicates were used in all analyses. In panels (a, b, c, e, h), densitometry of immunoblotted protein bands is plotted in the graphs in arbitrary units (a.u.). Data are reported as means ± *SEM*. Statistically significant differences are indicated (*p < 0.05, **p < 0.01). 100 µg/ml tocilizumab were applied in all experiments

In this context, we tested the effects of the neutralizing anti‐IL6 receptor antibody tocilizumab in HGPS cells. STAT3 phosphorylation was significantly inhibited in HGPS fibroblasts subjected to tocilizumab treatment (Figure 2a). In particular, while tocilizumab did not inhibit STAT3 phosphorylation on Tyrosine 705, phosphorylation of Serine 727, which is required for STAT3 transactivation activity (Wakahara et al., 2012), was significantly reduced by tocilizumab treatment (Figure 2a), which demonstrated its inhibitory activity in human dermal cells. Since IL6 activity is linked to hyperactivation of the DNA damage sensor γ‐H2AX, which is increased in HGPS cells, we hypothesized that inhibition of IL6 could reduce γ‐H2AX amount. Interestingly, in HGPS cells subjected to tocilizumab, significantly lower amount of γ‐H2AX was detected (Figure 2b).

Moreover, while HGPS fibroblast conditioned medium triggered γ‐H2AX even in normal human dermal fibroblasts, tocilizumab treatment significantly reduced HGPS secretome‐induced γ‐H2AX accumulation (Figure 2c). The latter observation showed the efficacy of the proposed treatment on the bystander effect of HGPS cellular secretome and in particular of secreted IL6. Importantly, in HGPS‐conditioned normal dermal fibroblasts, Senescence‐Associated beta‐Galactosidase (SA‐βGal) staining was significantly increased, while tocilizumab prevented SA‐βGal increase (Figure 2d). The latter result showed the inhibitory effect of tocilizumab on secretome‐induced cellular senescence. Consistently, in HGPS fibroblasts, tocilizumab treatment elicited downregulation of the senescence marker p21 (Figure 2e), which is upregulated in HGPS (Mattioli et al., 2018). These results suggested inhibition of cellular senescence and a general improvement of HGPS cellular phenotype upon tocilizumab treatment. In agreement with this observation, significant improvement in nuclear shape, measured by the contour ratio algorithm, was observed in HGPS fibroblasts treated with tocilizumab, while lamin A/C immunofluorescence signal was not significantly affected (Figure 2f). Moreover, chromatin organization was improved in tocilizumab‐treated cells, as also detectable from recovery of facultative heterochromatin areas (Figure 2f, arrowheads) (Lattanzi et al., 2007). Since nuclear dysmorphism has been directly correlated with the amount of progerin in the nuclear lamina (Columbaro et al., 2005), we decided to test progerin levels and localization in tocilizumab‐treated cells. Sharp progerin labeling was detected in untreated HGPS fibroblasts (Figure 2g). Interestingly, in HGPS cells subjected to tocilizumab, progerin fluorescence signal was diminished even in residual dysmorphic nuclei, where the mutated protein formed nuclear aggregates, while reduction in MFI was observed in the whole cell population (Figure 2g). Biochemical analysis confirmed significant reduction of progerin amount in HGPS fibroblasts subjected to tocilizumab treatment (Figure 2h). Although reduced lamin A and C amount was also detected in antibody‐treated cells, tocilizumab determined a significant increase in lamin A to progerin ratio (Figure 2h), which is relevant to the improvement of HGPS cellular phenotype (Pellegrini et al., 2015).

### 2.3 Effects of tocilizumab in muscle, tendons, and bone of *Lmna^G609G^* ^*/G609G*^ mice

**FIGURE 3:**
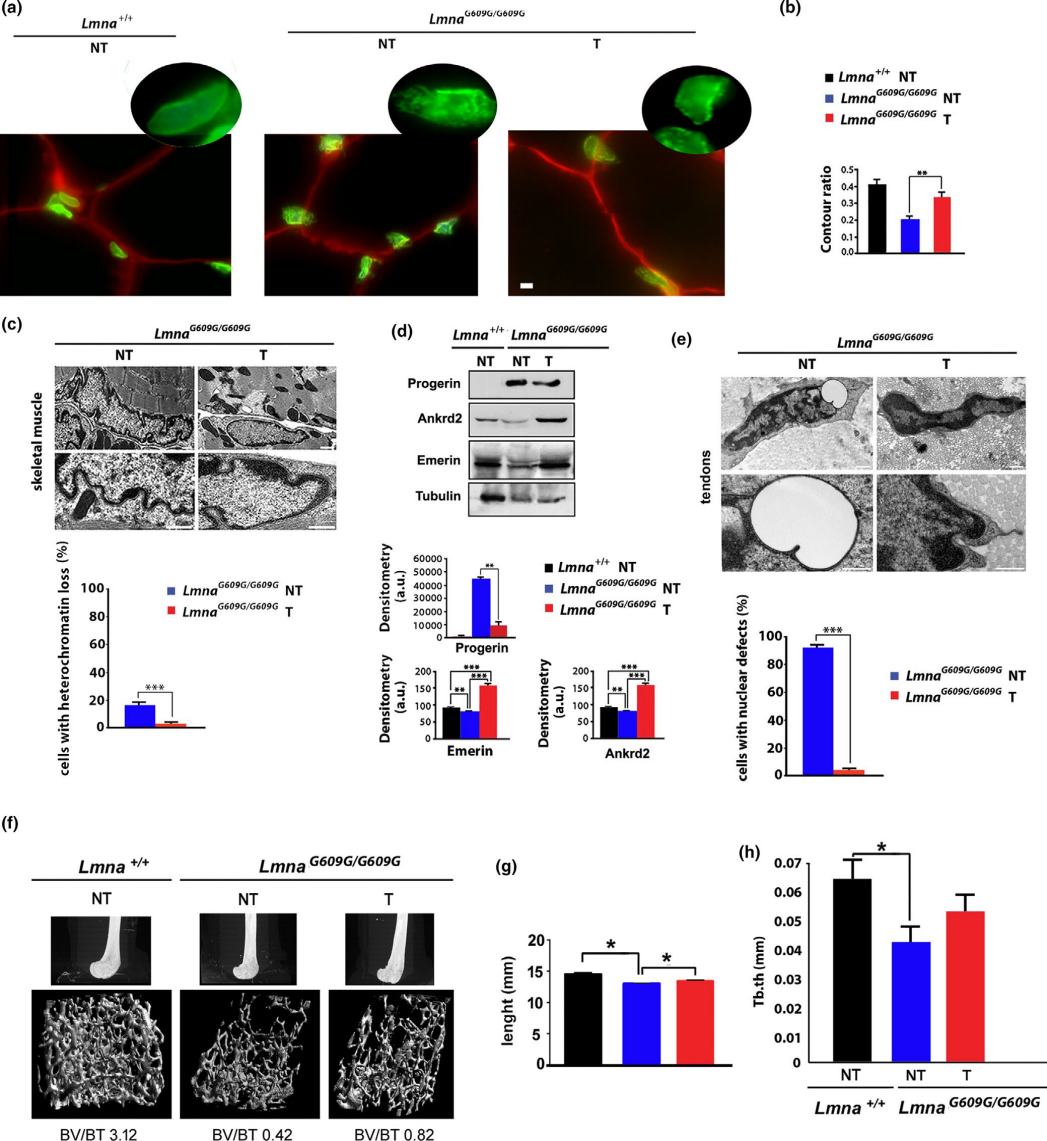
Effects of tocilizumab in muscle, tendons and bone of *Lmna^G609G^* ^/G609G^ mice. (a) Immunofluorescence staining of lamin A/C (green) in *Lmna* ^+/+^ or *Lmna^G609G^* ^/^ *^G609G^*mice (mean age 100 days), left untreated (NT) or treated (T) with tocilizumab. Muscle fibers are delineated by perlecan staining (red). Higher magnification of nuclei labeled by lamin A/C antibody is shown in the insets. Scale bars, 10 µm. (b) Contour ratio of muscle nuclei in tissue from three different *Lmna* ^+/+^ or *Lmna^G609G^* ^/^ *^G609G^* vehicle‐treated (NT) or tocilizumab‐treated mice (T). The analysis was performed in 100 nuclei per sample observed by electron microscopy. (c) Transmission electron microscopy analysis of skeletal muscle nuclei from vehicle‐treated (NT) or tocilizumab‐treated *Lmna^G609G^* ^/^ *^G609G^*mice (T). Scale bars, 1 µm. (d) Western blot analysis of progerin, Ankrd2 and emerin in muscle from *Lmna* ^+/+^ or *Lmna^G609G^* ^/^ *^G609G^* mice, vehicle‐treated (NT) or treated with tocilizumab (T). Tubulin was used as a loading control. Densitometry of immunoblotted protein bands is plotted in the graphs in arbitrary units (a.u.). (e) Transmission electron microscopy analysis of tendons from *Lmna^G609G^* ^/G609G^ mice subjected to vehicle (NT) or tocilizumab (T). Cell nuclei of *Lmna^G609G^* ^/G609G^ mouse tendons show vesicles in the perinuclear space (NT, round vesicles), which are not observed in tocilizumab‐treated mice. Heterochromatin areas appear also disorganized in progeroid mouse tendon nuclei, while recovery of heterochromatin at the nuclear periphery is observed in tocilizumab‐treated *Lmna^G609G^* ^/G609G^ mouse tissue. The analysis was performed in 50 nuclei per sample. Scale bars, 1 µm. (f) microCT scans of femur from *Lmna* ^+/+^, untreated *Lmna^G609G^* ^/^ *^G609G^* (NT) or tocilizumab‐treated *Lmna^G609G^* ^/^ *^G609G^* mice. Mean values of relative bone volumes (bone volume/tissue volume (BV/TV)) of the corresponding samples are indicated under each picture. (g) Mean femur biomechanical length (length) and (h) trabecular thickness values (Tb.th) measured in groups of three *Lmna* ^+/+^, untreated *Lmna^G609G^* ^/^ *^G609G^* (NT) or tocilizumab‐treated *Lmna^G609G^* ^/^ *^G609G^* mice (T). Three biological replicates were used in each experiment. Data are reported as means ± *SEM*. Statistically significant differences are indicated (*p < 0.05, **p < 0.01, ***p < 0.001). Mean age of mice (all males), 100 ± 6 days

Based on these results, we treated progeroid mice with tocilizumab starting at weaning (4 weeks of age) and evaluated antibody effects in various tissues known to be affected in this mouse model (Zaghini et al., 2020). Figure 3 shows the outcome of tocilizumab treatment in the musculoskeletal system of *Lmna^G609G^* ^/^ *^G609G^* mice. Muscle fibers did not show irregular shape in progeroid mice (Figure 3a). However, in skeletal muscle of vehicle‐treated *Lmna^G609G^* ^/^ *^G609G^*mice at 100 days of age, altered nuclear shape was observed by lamin A/C labeling of muscle cryosections (Figure 3a). Nuclear shape was significantly improved in muscle from age‐matched tocilizumab‐treated *Lmna^G609G^* ^/^ *^G609G^* mice, as measured according to the contour ratio algorithm in myonuclei observed by electron microscopy (Figure 3b,c). In fact, ultrastructural analysis showed severe nuclear morphological abnormalities in *Lmna^G609G^* ^/^ *^G609G^* mouse muscle consisting of nuclear envelope folding and loss of peripheral heterochromatin, which were recovered in muscle from tocilizumab‐treated progeroid mice (Figure 3c). On the other hand, nuclear positioning and sarcomere ultrastructural organization were not altered in progeroid mice (Figure 3a,c). Moreover, as observed in cultured HGPS cells, in vivo tocilizumab administration reduced progerin accumulation in *Lmna^G609G^* ^/^ *^G609G^* muscle tissue (Figure 3d). Noteworthy, in progeroid mouse muscle the lamin‐binding partner emerin was reduced, while tocilizumab restored emerin amount (Figure 3d). We further investigated the muscle‐specific oxidative stress responsive protein Ankrd2 (Cenni et al., 2019). In *Lmna^G609G^* ^/^ *^G609G^* mouse muscle, Ankrd2 amount was significantly reduced, while tocilizumab treatment increased Ankrd2 levels to a condition comparable to wild‐type mouse tissue (Figure 3d).

Motor function in progeria might be also affected by defects in tendons. By ultrastructural analysis, misshapen nuclei were also detected in tendons of *Lmna^G609G^* ^/^ *^G609G^* progeroid mice (Figure 3e). In particular, enlargement of perinuclear space with formation of vesicles was consistently observed in the vast majority of examined tendon nuclei (Figure 3e). These nuclear defects were abolished in mice subjected to tocilizumab (Figure 3e).

Then, we analyzed bone phenotype in *Lmna^G609G^* ^/^ *^G609G^* mice by microCT scan analysis and observed an altered structure of femur condyles (Figure 3f). In age‐matched tocilizumab‐treated progeroid mice, bone trabecular organization was improved (mean BV/BT 0,82 in tocilizumab‐treated vs 0.42 in untreated progeroid mice, Figure 3f). Moreover, femur biomechanical length, which was significantly reduced in *Lmna^G609G^* ^/^ *^G609G^*mice, increased in tocilizumab‐treated animals relative to untreated littermates (Figure 3g). However, trabecular thickness (Figure 3h) and other bone parameters were not significantly improved by tocilizumab treatment.

In cultured cells derived from *Lmna^G609G^* ^/^ *^G609G^*mouse muscle or bone, tocilizumab treatment also elicited positive effects. In progeroid mouse myoblasts, nuclear shape and chromatin organization were severely affected (Figure S2A). However, in myoblasts subjected to tocilizumab, nuclear shape was improved and a trend toward recovery of heterochromatin clusters was observed (Figure S2A). All these effects were related to decrease in progerin levels (Figure S2B). Moreover, as reported above for HGPS cells (Figure 2), in vitro tocilizumab administration elicited lower γ‐H2AX and p21 levels in progeroid mouse myoblasts (Figure S2B), suggesting rescue of DNA damage response and senescence pathways. Since previous studies had reported altered differentiation rate of laminopathic bone precursors (Avnet et al., 2011; Scaffidi & Misteli, 2008; Strandgren et al., 2015; Vidal et al., 2012), we established osteoblast cultures from *Lmna^G609G^* ^/+^ mouse bone and checked the differentiation rate in the presence or absence of tocilizumab. Higher differentiation rate was observed in *Lmna^G609G^* ^/+^ osteoblasts, compared to osteoblasts derived from *Lmna* ^+/+^ mouse bone (Figure S2C). However, tocilizumab treatment of progeroid osteoblasts restored a differentiation rate comparable to wild‐type cells, as determined by measuring Alizarin red staining of cell cultures at 21 days in differentiation medium (Figure S2C). These results indicated that progeroid myoblasts and osteoblasts feature cellular abnormalities independently of the whole organism condition, yet at least in part determined by hyperactivation of IL6. As a whole, data here reported show that tocilizumab counteracts progerin effects in cells from the musculoskeletal apparatus.

### 2.4 Improvement of cardiovascular phenotype in tocilizumab‐treated Lmna^G609G/G609G^mice

**FIGURE 4:**
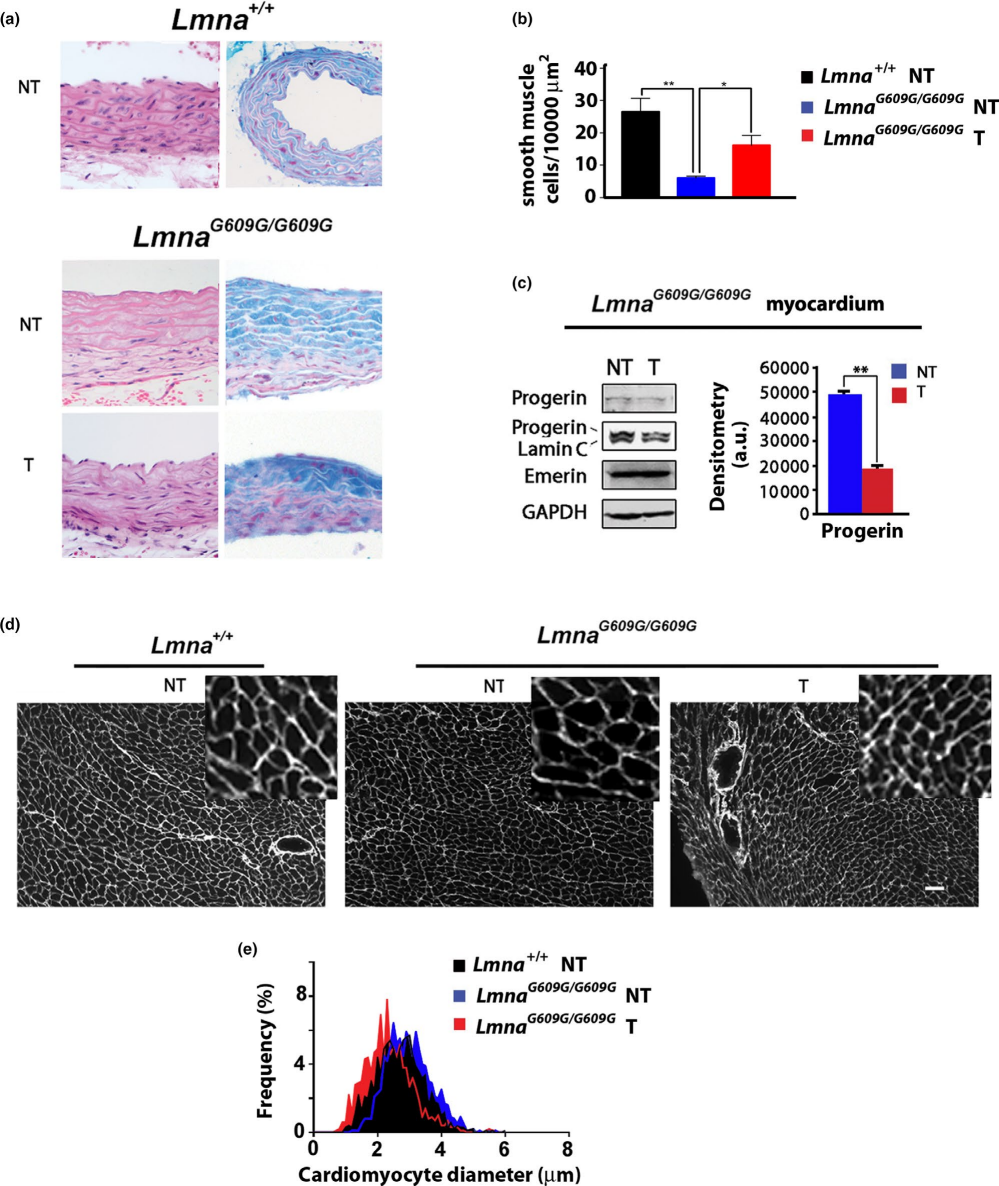
Tocilizumab reduces aorta lesions and cardiomyocyte hypertrophy in progeroid mice. (a) Aorta medium layer histochemical analysis in *Lmna* ^+/+^ and *Lmna^G609G^* ^/^ *^G609G^* mice left untreated (NT) or treated with tocilizumab (T). Hematoxylin–eosin (left panels) and Alcian Blue staining (right panels) show loss of cellularity and myxoid degeneration (accumulation of acidic mucopolysaccharides) in aorta from vehicle‐treated *Lmna^G609G^* ^/^ *^G609G^*mice (NT) and rescue with tocilizumab (T). (b) Mean number of smooth muscle cells detected in aorta sections is reported in the graph. (c) Western blot analysis of Progerin, Lamin C and Emerin in myocardium lysates from untreated (NT) or tocilizumab‐treated *Lmna^G609G^* ^/^ *^G609G^*mice (T). GAPDH bands are shown as loading controls. Densitometry of immunoblotted protein bands is plotted in the graphs in arbitrary units (a.u.). (d) Myocardium sections from 13 months old *Lmna* ^+/+^ or 3 months old *Lmna^G609G^* ^/^ *^G609G^* mice vehicle‐treated (NT) or treated with tocilizumab (T). Extracellular matrix surrounding cardiomyocytes was stained using an anti‐collagen VI antibody. Bar, 10 µm. (e) Graphs reporting diameter of cardiomyocytes in samples shown in (d). Three biological replicates were used. Data are reported as means ± *SEM*. Statistically significant differences are indicated (*p < 0.05, **p < 0.01)

Aorta is a main target of HGPS pathology (Hamczyk & Andres, 2019; Hamczyk, del Campo, et al., 2018; Hamczyk, Villa‐Bellosta, et al., 2018). Histological analysis of *Lmna^G609G^* ^/^ *^G609G^* mouse aorta sections showed a severe phenotype with loss of cellularity and myxoid degeneration (Figure 4a). In *Lmna^G609G^* ^/^ *^G609G^* mice, tocilizumab treatment reduced aorta degeneration (Figure 4a and Table S1).

As previously reported (Del Campo et al., 2019; Villa‐Bellosta et al., 2013), the number of smooth muscle cells was dramatically reduced in *Lmna^G609G^* ^/^ *^G609G^* mouse aorta (Figure 4b). However, tocilizumab administration led to a significant increase in smooth muscle cell number (Figure 4b). As observed in skeletal muscle, in vivo tocilizumab administration elicited reduction of progerin levels in myocardium, while lamin C and emerin amount were not changed (Figure 4c). In progeroid mice, we observed cardiomyocyte hypertrophy (Figure 4d). In fact, mean cross‐sectional diameter of cardiomyocytes from *Lmna^G609G^* ^/^ *^G609G^* mice aged 3 months was comparable to that measured in wild‐type littermates aged 13 months (Figure 4d), despite wild‐type mice weight reached 24 g, while *Lmna^G609G^* ^/^ *^G609G^*mice maximum weight was below 18 grams. In myocardium from tocilizumab‐treated *Lmna^G609G^* ^/^ *^G609G^* animals, cardiomyocyte mean diameter was significantly reduced, suggesting a less severe heart disorder (Figure 4d,e).

### 2.5 Improvement of adipose tissue phenotype in tocilizumab‐treated progeroid mice

**FIGURE 5:**
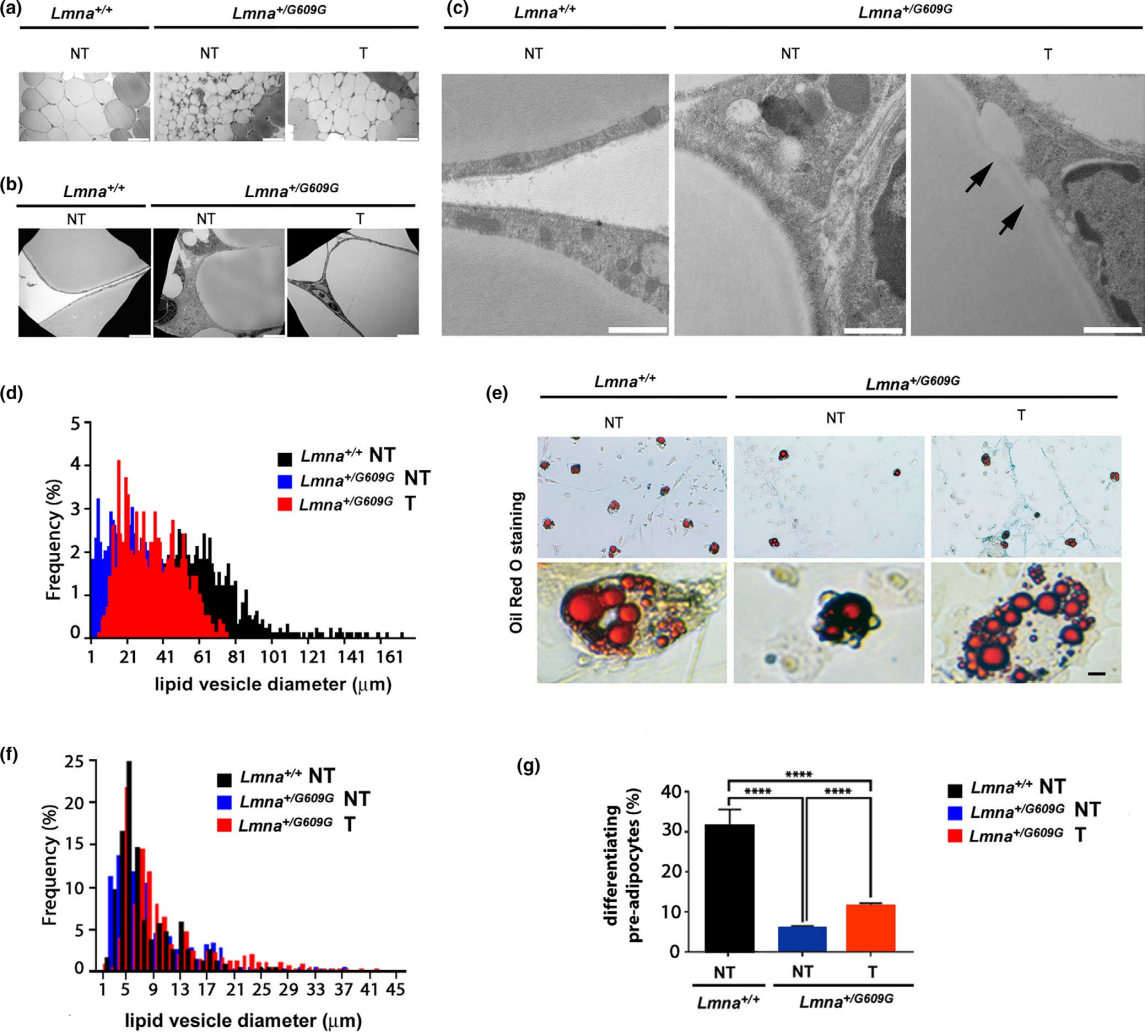
Tocilizumab improves adipose tissue phenotype in progeroid mice. (a) Light microscopy observation of semithin sections of subcutaneous adipose tissue from *Lmna* ^+/+^or *Lmna^G609G^* ^/+^ vehicle‐treated (NT) or tocilizumab‐treated mice (T). Semithin sections were obtained from epon resin‐embedded tissue prepared for electron microscopy analysis. (b, c) Electron microscopy analysis of adipose tissue samples shown in (a). Arrows indicate fusing lipid vesicle. (d) Quantitative analysis of adipocyte size in adipose tissue samples shown in (a–c). A trend toward wild‐type size distribution in tocilizumab‐treated *Lmna^G609G^* ^/+^mice is observed. (e) Oil red O staining of white pre‐adipocytes derived from *Lmna* ^+/+^ or *Lmna^G609G^* ^/+^ mouse subcutaneous fat. (f) Graph showing the distribution of lipid vesicle size. (g) Graph representing the percentage of differentiating pre‐adipocytes in cell cultures. Data are in arbitrary units (a.u.). Three biological replicates were used.Scale bars: (a), 100 µm; (b) 5 µm; (c), 1 µm, (e), 10 µm. Statistically significant differences are indicated (****p < 0.0001)

Fat loss is rapid in *Lmna^G609G^* ^/^ *^G609G^* mice, and almost all white adipose tissue is quickly lost around 5 weeks (Zaghini et al., 2020), so that evaluation of chronic treatment starting at weaning is not possible. Thus, to test the effect of tocilizumab on adipose tissue, we analyzed subcutaneous fat from control and tocilizumab‐treated *Lmna^G609G^* ^/+^ mice, which show slower progression of lipodystrophy, detectable at week 16 of age and progressing to complete atrophy of subcutaneous fat at week 43 (Zaghini et al., 2020). A dystrophic phenotype with high adipocyte diameter variability was observed in white fat from untreated progeroid mice aged 200 days (Figure 5a). In adipose tissue from tocilizumab‐treated *Lmna^G609G^* ^/+^mice, a more homogeneous adipocyte size and an overall increase in adipocyte mean diameter were observed (Figure 5a). Impaired terminal differentiation of adipocytes was observed by ultrastructural analysis in tissue from untreated *Lmna^G609G^* ^/+^mice (Figure 5b), while fusion of lipid droplets appeared to be increased in tocilizumab‐treated mouse adipose tissue (Figure 5c). As quantitative analysis confirmed the increase of adipocyte dimensions in white fat from tocilizumab‐treated mice (Figure 5d), we decided to test the effect of IL6 neutralization on adipocyte precursors. To this end, we established pre‐adipocyte cultures from *Lmna^G609G^* ^/+^ white adipose tissue and induced adipogenic differentiation (Pellegrini et al., 2019). Differentiation of *Lmna^G609G^* ^/+^ pre‐adipocytes was reduced, relative to *Lmna* ^+/+^ pre‐adipocytes, and significantly improved when tocilizumab was added to differentiation medium (Figure 5e), as demonstrated by enhanced lipid vesicle size (Figure 5f) and increased percentage of differentiating cells (Figure 5g).

### 2.6 Phenotype improvement in tocilizumab‐treated progeroid mice

**FIGURE 6:**
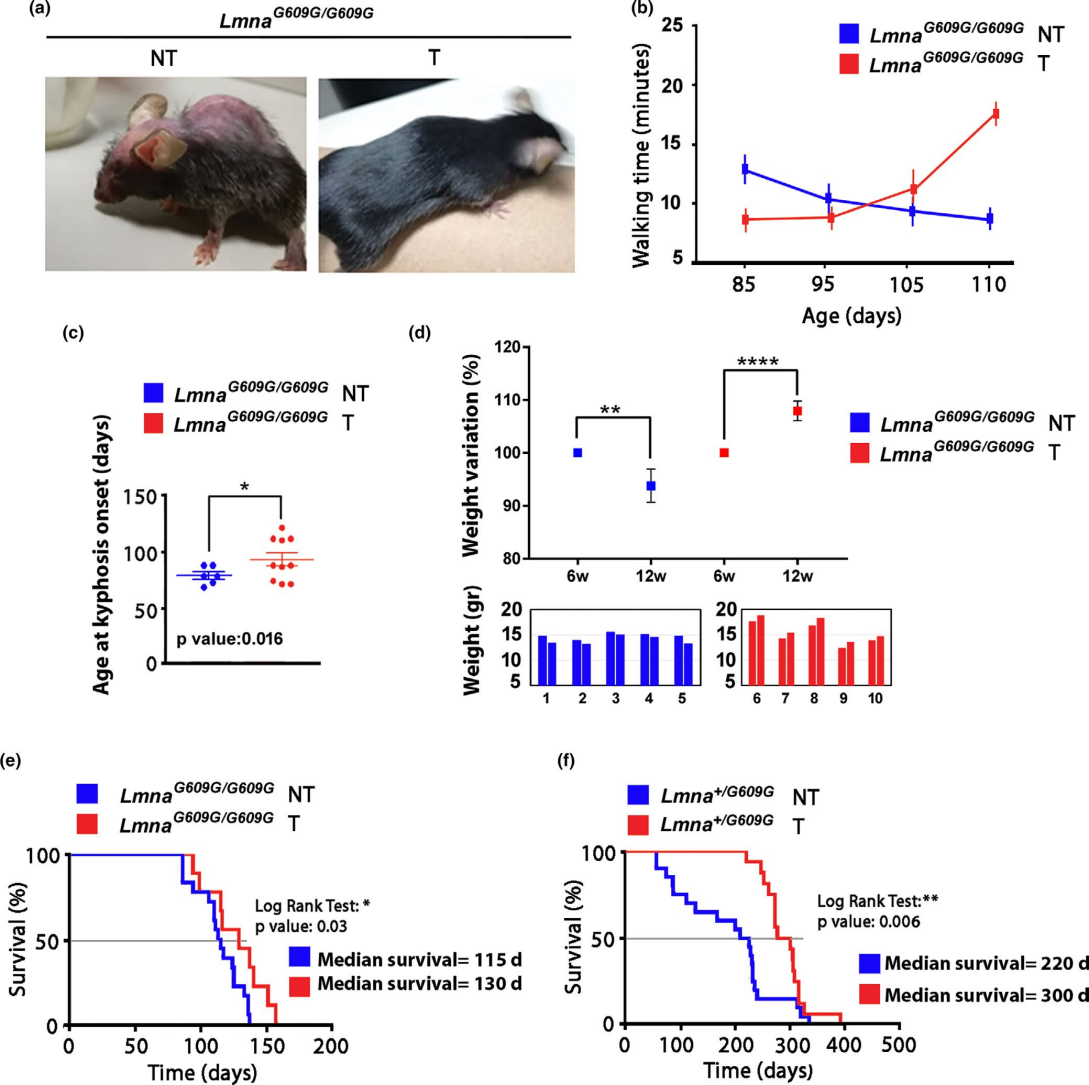
Tocilizumab improves progeroid phenotype and lifespan in progeroid mice. (a) Representative photographs of untreated (NT) and tocilizumab‐treated (T) *Lmna^G609G^* ^/^ *^G609G^* mice at 80 days of age. (b) Motility test of untreated (NT, *n* = 3, all males) and tocilizumab‐treated *Lmna^G609G^* ^/^ *^G609G^* mice (T, *n* = 3, all males). In the graph is reported the walking time (minutes) in a period of 30 min. Differences at 85 and 110 days are statistically significant (*p* < 0.01). (c) Age at kyphosis onset (days) of untreated (NT, *n* = 6, 3 females, 3 males) and tocilizumab‐treated (T, *n* = 7, 5 females, 2 males) *Lmna^G609G^* ^/^ *^G609G^* mice. Statistically significant difference is indicated (*p < 0.05). (d) Body weight variation (%) of untreated and tocilizumab‐treated *Lmna^G609G^* ^/^ *^G609G^* mice between 6 (6 w) and 12 weeks of age (12 w). Mean values measured in 5 mice per group (2 females, 3 males) are reported in the upper graph, weight referred to each animal (indicated as 1–10) is reported in the lower graphs (left bars, 6 weeks weight; right bars, 12 weeks weight). Statistically significant differences are indicated (**p < 0.01, ****p < 0.0001). (e) Kaplan–Meier survival plot showing the increase in life span of *Lmna^G609G^* ^/^ *^G609G^* mice treated with tocilizumab (*n* = 13, 7 females, 6 males) as compared with *Lmna^G609G^* ^/^ *^G609G^* untreated littermates (*n* = 18, 9 females, 9 males). *p* < 0.05, log‐rank/Mantel‐Cox test. (f) Kaplan–Meier survival plot showing the increase in life span of *Lmna^G609G^* ^/+^ mice treated with tocilizumab (*n* = 16, 9 females, 7 males) as compared with *Lmna^G609G^* ^/+^ untreated littermates (*n* = 20, 3 females, 17 males). *p* < 0.01, log‐rank/Mantel‐Cox test. The age at 50% survival (median survival) is indicated next to each graph (d, days)

By visual inspection, the phenotype of tocilizumab‐treated *Lmna^G609G^* ^/^ *^G609G^* mice appeared greatly improved with respect to their untreated littermates (Figure 6a). Alopecia was reduced, and the quality of the fur, which was typically altered in this mouse model (Zaghini et al., 2020), was better preserved (Figure 6a). Motor activity was also significantly improved by antibody treatment, as demonstrated by open field tests (Figure 6b). Even in very old mice, motor activity was preserved (Figure 6b and Movie S1). Moreover, the onset of kyphosis was delayed in tocilizumab‐treated mice (Figure 6c). Slowdown of weight loss was also determined by tocilizumab treatment. In fact, while all examined *Lmna^G609G^* ^/G609G^ mice showed significant weight loss between week 6 and 12 of their life, weight gain was observed in all tocilizumab‐treated progeroid mice in the same time frame (Figure 6d). A significant, though moderate, life span extension was obtained in *Lmna^G609G^* ^/^ *^G609G^* mice subjected to tocilizumab (Figure 6e), while more relevant increase in survival was determined in heterozygous mice (Figure 6f).

## 3 DISCUSSION

Cell intrinsic and cell‐independent mechanisms play a synergistic role in HGPS pathogenesis (Bidault et al., 2020; Del Campo et al., 2019; Cenni et al., 2020; Kreienkamp et al., 2016, 2018, 2019; Kreienkamp & Gonzalo, 2020; Osmanagic‐Myers et al., 2019). Thus, an efficient therapeutic strategy needs to counteract both mechanism types. Here, we confirmed increase of IL6 in the culture medium of HGPS fibroblasts and showed IL6 increase also in Mandibuloacral Dysplasia cells, suggesting that IL6 signaling could play a role in other *LMNA*‐linked progeroid laminopathies. We further showed that progerin expression is sufficient to activate both IL6 and NF‐kB promoters, demonstrating that the increase in IL6 is a transcriptional effect. Active IL6 signaling, demonstrated by nuclear accumulation of phosphorylated STAT3 as well as by the activation of DNA damage signaling elicited by the HGPS secretome in normal dermal fibroblasts, appeared to be relevant for HGPS pathogenesis. Thus, the aim of our research was to test the potential benefit of treatment with tocilizumab in HGPS preclinical models. Our results show that not only tocilizumab is able to reduce IL6 signaling in HGPS cells, but elicits reduction of progerin levels with rescue of nuclear defects caused by progerin accumulation. Particularly relevant is the recovery of chromatin organization observed in mouse tissues upon in vivo administration of tocilizumab, which shows that even early cell intrinsic pathogenetic effects are rescued by the antibody. A plethora of studies have shown that progerin, as any farnesylated prelamin A form, affects nuclear morphology and chromatin organization (Filesi et al., 2005; Lattanzi et al., 2007; Pellegrini et al., 2015). Thus, rescue of nuclear defects in tocilizumab‐treated cells and tissues is an obvious consequence of reduced progerin accumulation. In previous studies, stress‐induced wild‐type prelamin A increase has been demonstrated (Lattanzi et al., 2014; Mattioli et al., 2018; Ragnauth et al., 2010). It appears likely that stress conditions contribute to enhanced progerin levels in HGPS cells, while reduced stress signaling elicited by inhibition of IL6 could reduce stress‐dependent progerin increase. However, a relationship between progerin levels and IL6 activity has been reported in previously published studies and tocilizumab effect on progerin levels could depend on interconnected cellular pathways involving JAK/STAT signaling (Liu et al., 2019). On the other hand, STAT1‐mediated inflammatory response, triggered by replication fork stalling and damaged DNA accumulation in the cytoplasm, has been demonstrated in HGPS cells and a synergistic effect with STAT3‐related IL6 signaling on progeroid phenotype appears likely (Kreienkamp et al., 2018). Interestingly, any drug able to counteract progerin accumulation has been reported to attenuate STAT1 activity (Kreienkamp et al., 2018), reinforcing the view of a direct link between progerin levels and inflammatory response.

In vivo tocilizumab administration to *Lmna^G609G^* ^/^ *^G609G^*progeroid mice allowed us to show some tissue‐specific effects. Tocilizumab elicited positive effects in skeletal muscle, including rescue of the muscle‐specific stress responsive factor Ankrd2 (Cenni et al., 2019), which was decreased in progeroid muscle. This result is particularly relevant, as resolution of oxidative stress and inflammation could be fostered by Ankrd2‐dependent NF‐kB inhibition (Bean et al., 2014), with potential effects on IL6 levels and other NF‐kB effectors of inflammatory signaling. Reduction of emerin in the skeletal muscle of progeroid mice is a novel finding, which could suggest defects in skeletal muscle regeneration associated with progerin expression (Capanni et al., 2009; Squarzoni et al., 2005). Emerin is the main lamin A/C binding partner, known to play a major role in striated muscle and linked to Emery–Dreifuss muscular dystrophy. We previously observed that emerin localization at the nuclear membrane is related to its interplay with prelamin A (Capanni et al., 2009), a mechanism that could be impaired in the presence of a defective prelamin A form as progerin. Of note, concomitant with progerin decrease, emerin levels are rescued in skeletal muscle from tocilizumab‐treated *Lmna^G609G^* ^/^ *^G609G^* mice, while emerin levels are not altered in myocardium of progeroid mice nor they are affected by tocilizumab in heart tissue. Further, emerin levels are not altered in HGPS fibroblasts, again suggesting a tissue‐specific deficiency. Given the role of emerin and emerin–prelamin A interplay in muscle physiology and pathology (Capanni et al., 2008, 2009; Squarzoni et al., 2005), deeper understanding of emerin fate in HGPS skeletal muscle might provide new insights not only into HGPS pathogenesis, but also on aging‐associated muscle disorders as sarcopenia. Overall, altered nuclear structure and disorganized chromatin here observed both in muscle and tendons, as well as altered expression levels of emerin and Ankrd2 in skeletal muscle, indicate an insofar unrecognized condition that might contribute to motor function impairment in HGPS (Levy et al., 2018).

A limitation of our study is lack of electrophysiological evaluation of heart functionality. However, we focused on tissue morphology and were able to demonstrate improvement of aorta lesions, including smooth muscle cell loss, and amelioration of cardiomyocyte hypertrophy in myocardium from tocilizumab‐treated mice. Hypertrophy of myocardium was previously observed in mouse models expressing endothelium‐targeted progerin (Osmanagic‐Myers et al., 2019; Sun et al., 2020). As SASP activation including IL6 hypersecretion was determined in one of those progeroid mouse strains, it is likely that cell extrinsic mechanisms contribute to hypertrophy of cardiac tissue (Sun et al., 2020), an hypothesis supported by our results.

Lipodystrophy is a prominent phenotype in all progeroid laminopathies (Cenni et al., 2018). None of currently available drugs was able to counteract adipose tissue loss, either in less severe *LMNA*‐linked lipodystrophies (Araujo‐Vilar & Santini, 2019) or in HGPS (Gordon et al., 2018). On the other hand, in *Lmna^G609G^* ^/^ *^G609G^* mice subjected to high fat diet, an impressive life span extension was obtained (Kreienkamp et al., 2019; Kreienkamp & Gonzalo, 2020), suggesting a main role of adipose tissue loss in HGPS pathogenesis. Thus, amelioration of white adipose tissue condition by tocilizumab might contribute to the overall improvement of health status here observed in progeroid mice. An application of the antibody to other laminopathies featuring lipodystrophy warrants investigation.

Bone phenotype (Gargiuli et al., 2018) was also improved by tocilizumab treatment. In fact, increased femur biomechanical length and partial rescue of altered condyle trabecular structure along with delay in the onset of kyphosis were observed in antibody‐treated animals. Our results obtained in cultured *Lmna^G609G^* ^/^ *^G609G^* osteoblasts confirmed the aberrantly increased differentiation rate of laminopathic osteoblasts previously demonstrated in bone progenitor cells carrying the human progeria G608G *LMNA* mutation (Scaffidi & Misteli, 2008) and Mandibuloacral dysplasia osteoblasts (Avnet et al., 2011). We cannot rule out the possibility, suggested in other studies, that osteoblast activity could be instead decreased in progeroid mice in different bone districts (Strandgren et al., 2015). However, here we show a differentiation rate comparable to wild‐type cells in progeria osteoblasts subjected to tocilizumab, suggesting potential rescue of normal bone turnover. Since IL6 is an osteoclastogenic cytokine and tocilizumab has been shown to reduce the RANKL/OPG ratio, which regulates osteoclastogenesis (Kamiya et al., 2019), we predict that antibody administration to *Lmna^G609G^* ^/^ *^G609G^* mice might also reduce osteoclastogenesis by directly targeting the NF‐kB/RANKL pathway. Along this line, a recent study using another IL6‐neutralizing antibody showed amelioration of osteoporosis associated with hypersecretion of IL6 due to *LMNA* deficiency (Xiong et al., 2020).

A relevant outcome of tocilizumab treatment was the amelioration of motor activity in progeroid mice. Open field tests showed better performance of antibody‐treated mice even at advanced age. Although skeletal abnormalities and motor impairment do not appear as life‐threatening features of progeria, they severely impact on patient quality of life as children affected by progeria can only walk short distances due to articular impairment (Gordon et al., 2018). In this respect, it is worth to remind that tocilizumab is currently used to treat rheumatoid arthritis, where the antibody is expected to reduce IL6 inflammatory activity affecting articular cartilage (Mihara et al., 2011).

A main bias in this and almost all previously published studies conducted in progeroid mice is failure to identify an obvious cause of premature death (Hamczyk & Andres, 2019). The moderate increase in life span obtained by tocilizumab administration suggests that pathogenetic pathways specifically relevant to animal survival were not rescued. For instance, despite amelioration of adipose tissue turnover and attenuation of IL6 signaling, metabolic effects related to dysregulation of other cytokines (Bidault et al., 2020; Griveau et al., 2020; Liu et al., 2019) might suddenly establish a fatal condition. Optimization of tocilizumab dosage and combination with drugs or molecular approaches already explored for HGPS treatment may pave the way to effective therapeutic strategies (Cenni et al., 2020; Liu et al., 2019). For instance, tocilizumab could elicit a synergistic effect with lonafarnib, the farnesyltransferase inhibitor used in the ongoing HGPS clinical trial (https://www.clinicaltrials.gov/ct2/show/NCT00425607). Of note, lonafarnib has been reported to lower progerin levels in HGPS iPSCs‐derived smooth muscle cells subjected to biomechanical strain (Ribas et al., 2017). Also, combination with statins, shown to improve HGPS cellular phenotype (Columbaro et al., 2005) and reduce IL6 levels (Ribas et al., 2017), warrants investigation.

Finally, our study indicates that tocilizumab could be explored in aging‐associated disorders, including sarcopenia, cachexia, and motor impairment and more in general to mitigate the detrimental effects of age‐related inflammation that impinge upon the onset of dysfunctions and disability at late ages and on the overall quality of life in the elderly.

## 4 MATERIALS AND METHODS

### 4.1 IL6 detection and neutralization

Tocilizumab, a monoclonal anti‐IL6R neutralizing antibody (Mihara et al., 2011), was from Roche. IL6 levels in culture media of control and HGPS fibroblasts were measured by ELISA using kits from RD‐Systems (Human IL‐6 Quantikine HS ELISA Kit) following manufacturer's instructions. A fluorescent plate reader (Infinite M200‐6110; Tecan) was used to measure IL6 signal. For IL6 neutralization in HGPS fibroblast cultures, 100 µg/ml of neutralizing antibody was added to culture medium for 72 h. Tocilizumab was administered to *Lmna^G609G^* ^/^ *^G609G^* or *Lmna^G609G^* ^/+^ by intraperitoneal injection every three days starting at weaning (typically at post‐natal week 4) and continued up to the humane endpoint, when the animals were euthanized. A dosage of 40 mg/kg (body weight) per week was estimated the most efficient. Saline solution was injected in parallel to animals to be used as controls.

### 4.2 Mice

All animal studies were performed in accordance with EU regulations, the guidelines of the Italian Ministry of Health and the local committee for animal welfare. Experiments were also in compliance with ethical rules and the experimental protocol was approved by the Italian Ministry of Health (No. 653/2016‐PR issued on 07/04/2016 and update No. AC750.13‐1105). Homozygous or heterozygous mice (C57BL/6 strain) carrying the mouse *Lmna* G609G mutation, equivalent to *LMNA* G608G mutation of human HGPS, were kindly provided by Prof. Carlos Lopez‐Otin (Oviedo University, Spain) (Osorio et al., 2012).

Phenotype and molecular features of *Lmna^G609G^* ^/^ *^G609G^* and *Lmna^G609G^* ^/+^ progeroid mice have been characterized and previously reported (Osorio et al., 2011; Zaghini et al., 2020). Co‐housed wild‐type littermates (*Lmna* ^+/+^) were used as controls. Two to five mice were housed in each cage, at a constant temperature of 22 ± 1°C under a 12‐hour light/12‐hour dark cycle with free access to food and water. Animals were randomly assigned to each group. Analyses of phenotypes of mice were performed at different times throughout their lifespan, and at time of death. Procedures performed in mice include: weight monitoring), performance tests to assess locomotor activity (open field test), micro‐computed tomography (microCT) analysis of femurs with a microCT scan and tissue collection. As a whole, 10 *Lmna* ^+/+^male mice and 10 females, 22 *Lmna^G609G^* ^/+^ males and 20 females and 13 *Lmna^G609G^* ^/^ *^G609G^* males and 7 females were used in this study.

To assess motor activity, a subset of mice (3 mice per genotype, all males) underwent the open field test at 85, 95, 105 and 110 days of age. A semi‐transparent plastic white rectangular box (70 cm × 50 cm), was used as arena (Zaghini et al., 2020). At the beginning of the test, each mouse was set in the middle of the arena. Experienced operators during a 30 min period observed and recorded and evaluated: travelled quadrants, time spent moving, time spent in the central area, and vertical movements. The room was isolated from sound, and unintentional interruptions were avoided.

### 4.3 Skeletal microCT

Mouse femurs immersed in saline solution within a plastic holder were analyzed on a microCT model Skyscan 1072 (Bruker Corp., MicroCT unit). The scanning parameters were set at a voxel resolution of 10.78 µm, 50 kV, 200 µA, 1 mm aluminum filter, exposure time 5936 ms, image averaged on 2 frames, rotation 180° and a rotation step of 0.9°. Tomographic image reconstruction was based on NRecon software (Bruker Corp., MicroCT unit). A global threshold was applied to select bone tissue (gray threshold value 117/255). Mouse femur and trabecular bone were also qualitatively analyzed on 3D reconstructions thanks to CT‐Vox software (Bruker Corp., MicroCT unit) to identify any possible alteration. Trabecular and cortical morphology of the femur were investigated by use of CT‐Analyzer software (Bruker Corp., MicroCT unit).Trabecular bone was measured on the following parameters: bone volume fraction (bone volume/total volume or BV/TV, %), trabecular bone thickness (TbTh, mm), trabecular separation (TbSp, mm), and trabecular bone number (TbN, 1/mm). Volume of interest for trabecular tissue was selected with the anatomical reference of the distal growth plate, starting about 0.215 mm proximally from the growth plate level (offset of 50 image slices), and extending in the direction of the femoral head for about 1.72 mm (height of 450 image slices. This section of the diaphysis is defined in coincidence to the length investigated by mechanical testing. The femur biomechanical length was defined as the longitudinal distance between the cranial side of the intertrochanteric fossa and the intercondylar fossa.

### 4.4 Cell cultures and transfection

All human cell cultures used in this study were from BioLaM biobank (Rizzoli Orthopedic Institute Ethical Committee approval no. 0018250–2016, in compliance with all local and EU ethical rules). Control and HGPS fibroblast cultures had been obtained from skin biopsies as previously described (Columbaro et al., 2005). Necropsy of *Lmna* ^+/+^, *Lmna^G609G^* ^/^ *^G609G^*, and *Lmna^G609G^* ^/+^progeroid mice was performed according to local and EU ethical rules. Cells were cultured in high‐glucose Dulbecco's modified Eagle's medium (DMEM) (D5648; Sigma) supplemented with 20% fetal bovine serum (FBS) (10270‐106; Thermo Fisher), 100 IU/ml penicillin and 100 µg/ml streptomycin (15140122; Thermo Fisher) (growth medium) in a 5% CO_2_ humidified atmosphere at 37°C.

Adipocyte, osteoblast, tenocyte and myoblast cultures were established as previously described from mouse subcutaneous fat, rib bone and skeletal muscle (vastus lateralis), respectively, and differentiated according to established protocols (Antoniel et al., 2020; Avnet et al., 2011; Mattioli et al., 2011; Pellegrini et al., 2019).

Differentiation of cultured mouse pre‐adipocytes was assessed by Oil Red O staining. Cells were washed twice with PBS and fixed with 10% Formalin Solution (HT501128, Fisher) in distilled water for 45 min. After a 5 min incubation with 60% isopropanol, Oil Red working solution was added to the fixed cells for 5 min at room temperature. Images were obtained by using a Zeiss Axio A1 inverted microscope equipped with a digital camera and ZEN software.

Mineralized matrix formation in osteoblast cultures was detected by alizarin red S (ARS) staining. Cells were washed twice with PBS and fixed with 10% Formalin Solution in disttilled water for 5 min, washed twice with distilled water and stained with ARS working solution for 5 min. Samples were observed using a Zeiss Axio A1 inverted microscope equipped with a digital camera. Pictures were taken using ZEN software.

To measure nuclear circularity, the contour ratio algorithm was used and calculated by following the formula:

$$\text{contour}\;\text{ratio}=4\pi \times \text{nuclear}\;\text{area}/\text{nuclear}\;{\text{perimeter}}^{2}$$

HEK293 cells were transiently transfected with plasmids expressing wild‐type prelamin A (LA‐WT), which undergoes normal maturation, progerin (LA‐Δ50), which cannot be processed by ZMPSTE24 endoprotease, or R527H‐mutated lamin A, associated with MADA (Lattanzi et al., 2007). Transfections were performed using lipofectamine‐2000 (18324012; Invitrogen) according to the manufacturer's instructions. After transfection, cells were incubated for 48 h, if not differently stated.

### 4.5 Biochemical analysis

Cells were fixed in 4% paraformaldehyde, post‐fixed using absolute methanol for 5 min, and stained according to previously published protocols (Mattioli et al., 2011). Primary antibodies were applied overnight at 4°C, and secondary antibodies were used for 1 hour at room temperature. Nuclei were counterstained with 4,6‐diamidino‐2‐phenylindole (DAPI). Sample observation and image acquisition were performed using a Nikon Eclipse Ni epifluorescence microscope equipped with a digital CCD camera and NIS‐ Elements 4.3 AR software. Photoshop CS and Photoshop 7 were used for image processing. Mean fluorescence intensity (MFI) was measured using NIS‐ Elements 4.3 AR.

For Western blot analysis, tissues and cells were lysed in buffer containing: 20 mM Tris‐HCl (pH = 7.5), 1% SDS, 1 mM Na3VO4, 1 mM PMSF, 5% beta‐mercaptoethanol, and protease inhibitors. Proteins were subjected to SDS gradient gel (5%–20%) electrophoresis and transferred to nitrocellulose membrane overnight at 4°C. After incubation with primary and secondary antibodies, immunoblotted bands were revealed by Invitrogen ECL detection system. Densitometry was performed by a Bio‐Rad GS800 Densitometer equipped with Quantity One Software. Densitometric values were normalized to corresponding GAPDH bands if not differently stated.

### 4.6 Histology

Skeletal muscle or myocardium fragments from *Lmna* ^+/+^ or *Lmna^G609G^* ^/^ *^G609G^*mice were frozen in melting isopentane and stored in liquid nitrogen. Unfixed cryosections were subjected to immunofluorescence staining as detailed above.

Samples of aortic arch promptly after necropsy were fixed with formalin and embedded in paraffin. Histology was based on hematoxylin–eosin, PAS stain (Bio Optica, Milan, Italy; P.A.S. Hotchkiss – MC Manus, 04‐130802) and Alcian stains at pH 2,5 and 1 (Bio Optica; Alcian Blu, 04‐160802). Reduction of cellularity in aorta middle coat was graded as described (Zaghini et al., 2020) and reported in Table S1. For aorta examination, three photographs per sample were acquired with a DFK 33UX264 camera coupled with a Leica TM DMLB microscope (TIFF format 2448 × 2048, Obj 20×).

The area was manually delineated (lowest selected area = 40,000 µm^2^), and leiomyocytes were manually counted with ImageJ software (https://imagej.nih.gov/ij/index.html/). Finally, cellularity was assessed and expressed as the number of leiomyocytes/10,000 µm^2^.

### 4.7 Antibodies

Antibodies employed in this study were as follows: anti‐STAT3 phosphorylated on tyrosine 705 (P‐STAT3 Y705), anti‐STAT3 phosphorylated on Serine 727 (P‐STAT3 S727), and anti‐STAT3 from Thermo Fisher Scientific; anti‐γ‐H2AX, rabbit polyclonal (4937; Cell Signaling); anti‐p21, rabbit polyclonal (MA5‐14949; Invitrogen); anti‐Emerin, mouse monoclonal (MONX10804; Monosan); anti‐β‐tubulin; anti‐lamin A/C, goat polyclonal (SC‐6215; Santa Cruz Biotechnology); anti‐Progerin, mouse monoclonal (13A4; Enzo); anti‐GAPDH, mouse monoclonal (MAB374; Millipore); anti‐Ankrd2, mouse monoclonal, clone YAS11 (LS‐Bio); and anti‐collagen VI, monoclonal (Millipore).

### 4.8 Transmission Electron Microscopy

Tissue fragments (tendon, skeletal muscle and adipose tissue) were fixed with 2.5% glutaraldehyde in 0.1 M cacodylate buffer, pH 7.3. Semithin sections were stained with toluidine blue for preliminary optical microscopy sample observation. Ultrathin sections were treated for transmission electron microscopy observation as described (Filesi et al., 2005). After post‐fixation with 1% osmium tetroxide in 0.1 M cacodylate buffer for 1hour, samples were dehydrated in an ethanol series, infiltrated with propylene oxide, and embedded in Epon812 epoxy resin following standard procedures. Ultrathin sections (60 nm thick) were stained with uranyl acetate and lead citrate and observed at a 0° tilt angle with a JEOL JEM‐1011 transmission electron microscope operated at 100 kV.

### 4.9 Luciferase assays

One day before transfection, human control or HGPS fibroblasts were seeded on 6‐well plates and transfected with 500 ng of luciferase reporters driven by the −2,161 to −41 bp IL6 promoter fragment (kindly provided by W. L. Farrar, NCI‐Frederick Cancer Research and Development Center, USA, Papi et al., 2012). Firefly Luciferase was normalized by co‐transfecting 10 ng of Thymidine Kinase Renilla Luciferase reporter (Promega Corporation). All luciferase assays were performed in triplicate following manufacturer's instructions (Promega).

### 4.10 Real‐time PCR

Total RNA was extracted using the TRI Reagent Solution (Invitrogen) and treated with TURBO DNase (Invitrogen). cDNAs were produced using the High‐Capacity RNA‐to‐cDNA Kit (Applied Biosystems) according to the manufacture's protocol. Gene expression was determined by qPCR and Power SYBR Green PCR master mix (Applied Biosystems). Expression analysis was performed using the Applied Biosystem 7900HT real‐time PCR system. Fold change of expression levels was analyzed by the ∆∆CT method, and transcript levels were normalized by using the housekeeping reference gene GAPDH. The qPCR primer list is reported in Table S2.

### 4.11 Statistical analysis

Statistical analysis was performed with GraphPad Prism version 7 (Prism). Data were expressed as means ± standard error of the mean (*SEM*), as indicated in the figure legends, and tested using one‐way ANOVA (for multiple comparisons) or two‐tailed Student's *t* test (two groups). For the comparison of different groups in Kaplan–Meier survival plots, we used a log‐rank (Mantel‐Cox) test. *p* values of ≤0.05 are considered statistically significant. *p < 0.05; **p < 0.01; ***p < 0.001, ****p < 0.0001; Non‐significant (NS), *p* ≥ 0.05.

## CONFLICT OF INTERESTS

None declared.

## AUTHOR CONTRIBUTIONS

SS and GL involved in conceptualization. ES, PS, EM, CC, VC, MRDA, DA, CB, MS, VP, AF, and GS involved in investigation. GL contributed to manuscript writing. GS, FB, MB, SS, and AZ contributed to supervision. GL involved in funding acquisition. AZ and GL contributed to resources.

## Supporting information

Fig S1

Fig S2

Video S1

Appendix S1

## Data Availability

The data that support the findings of this study are available from the corresponding author upon reasonable request.
