# Supplementary figures and images for: Interleukin‐6 neutralization ameliorates symptoms in prematurely aged mice
Source: Aging Cell. 2021 Jan 3;20(1):e13285. doi: 10.1111/acel.13285 (PMC7811841; doi:10.1111/acel.13285)

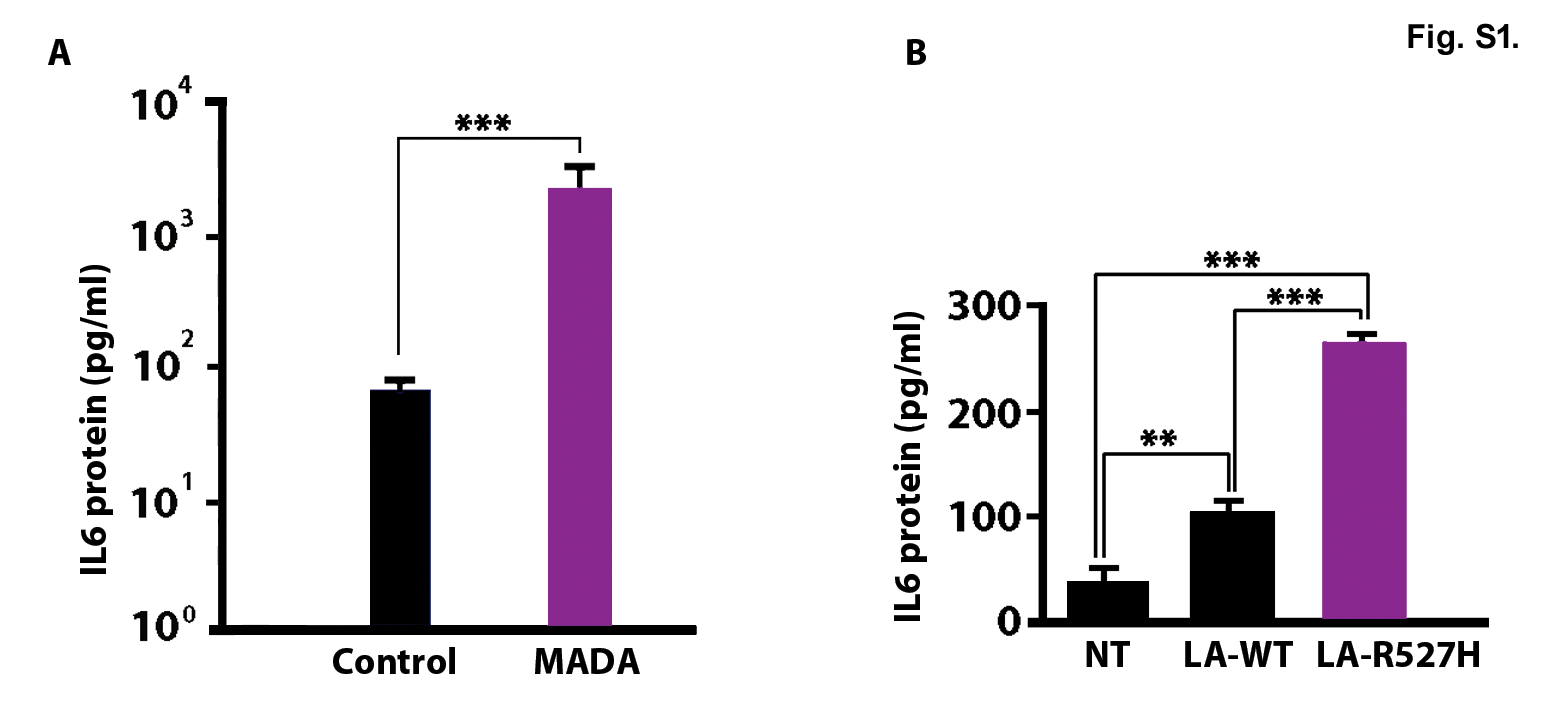

Supplement: Supplementary file 1 — Fig S1 [file ACEL-20-e13285-s001.tif]

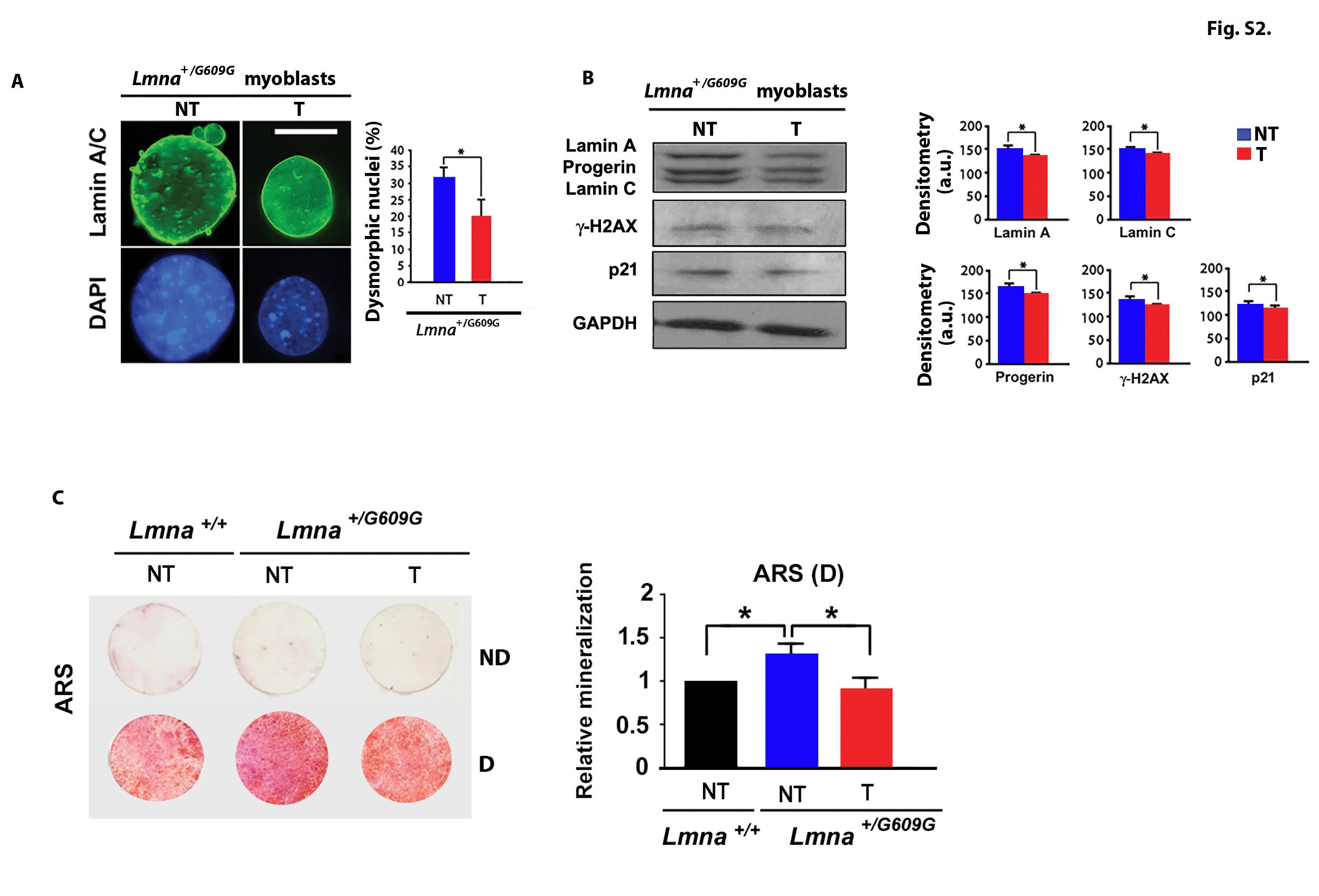

Supplement: Supplementary file 2 — Fig S2 [file ACEL-20-e13285-s002.tif]
