# Supplementary material for: Interleukin‐6 neutralization ameliorates symptoms in prematurely aged mice
Source: Aging Cell. 2021 Jan 3;20(1):e13285. doi: 10.1111/acel.13285 (PMC7811841; doi:10.1111/acel.13285)
Supplement: Supplementary file 4 — Appendix S1 [file ACEL-20-e13285-s004.pdf]

SUPPLEMENTARY MATERIALS

The supplementary materials include:

Fig. S1. IL6 secretion is increased in Mandibuloacral dysplasia cells.

Fig. S2. Tocilizumab effects in cultured *Lmna*$^{G609G/+}$ mouse myoblasts.

Table S1. Aorta grade of tissue degeneration in tocilizumab-treated *Lmna*$^{G609G/G609G}$ mice.

Table S2. List of primers used in this study.

Movie S1. Motor function is preserved in tocilizumab-treated *Lmna*$^{G609G/G609G}$ mice.

Supplementary figures

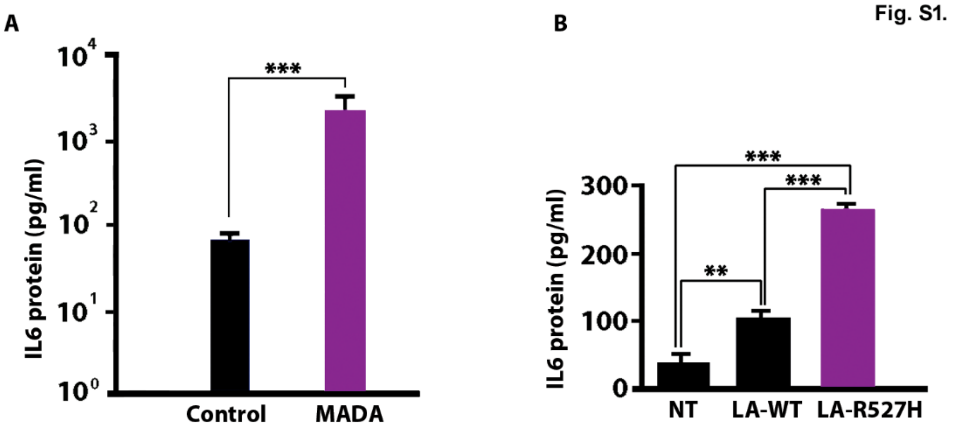

Fig. S1. IL6 secretion is increased in Mandibuloacral dysplasia (MADA) cells. *(***A***)* IL6 secretion in culture media of control (Control) and type A Mandibuloacral dysplasia (MADA) fibroblasts. MADA fibroblasts here analyzed carry the *LMNA*-R527H mutation in homozygosis (Filesi et al., 2005). (**B**) IL6 secretion in culture media of HEK293 not transfected (NT) or transiently transfected with plasmid carrying WT-*LMNA* (LA-WT) or R527H *LMNA* mutation (LA-R527H).

Fig. S2.

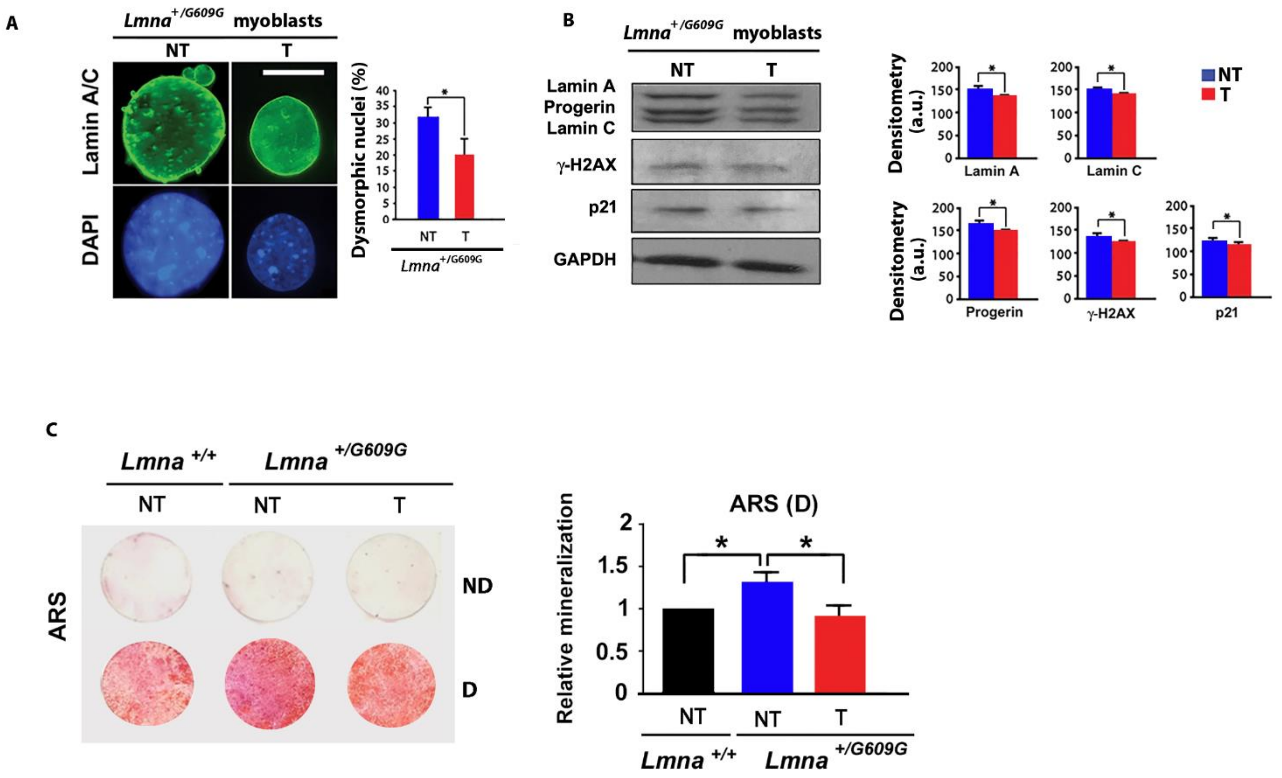

Fig. S2. Tocilizumab effects in cultured *Lmna*$^{G609G/+}$ myoblasts. Myoblast cell cultures were obtained from vastus lateralis of 200 days old mice. (**A**) Immunofluorescence analysis of Lamin A/C (green) in *Lmna*$^{G609G/+}$myoblasts (passage 2). Cells were left untreated (NT) or treated with 100μg/ml of tocilizumab (T). DAPI staining (blue) labels cell nuclei. Scale bar, 10 μm. (**B**) Western blot analysis of Progerin, Lamin A/C, γ-H2AX and p21 in *Lmna*$^{G609G/+}$ cultured myoblasts. Cells were left untreated (NT) or treated with tocilizumab (T). GAPDH bands are shown as protein-loading controls. Densitometry of immunoblotted protein bands is reported in the graphs in arbitrary units (a.u.). (**C**) Alizarin Red S (ARS) staining of osteoblasts derived from *Lmna*$^{G609G/+}$ mouse ribs at basal condition (ND) or differentiated (D), left untreated (NT) or treated with tocilizumab (T). Relative mineralization values (relative to untreated *Lmna*$^{+/+}$ osteoblast optical density). Three biological replicates were used in each experiment. Statistically significant differences are indicated (* $p < 0.05$). Cells were obtained from two *Lmna*$^{G609G/+}$mice, all males.

SUPPLEMENTARY TABLES

Table S1.

Lmna$^{G609G/G609G}$					
**treatment**	NT	NT	NT	NT	NT
**Age (days)**	105	109	109	114	108
**grade**	3	4	2	3	4
Lmna$^{G609G/G609G}$					
**treatment**	Tocilizumab	Tocilizumab	Tocilizumab	Tocilizumab	Tocilizumab
**Age (days)**	94	99	109	140	151
**grade**	2	2	2	3	4

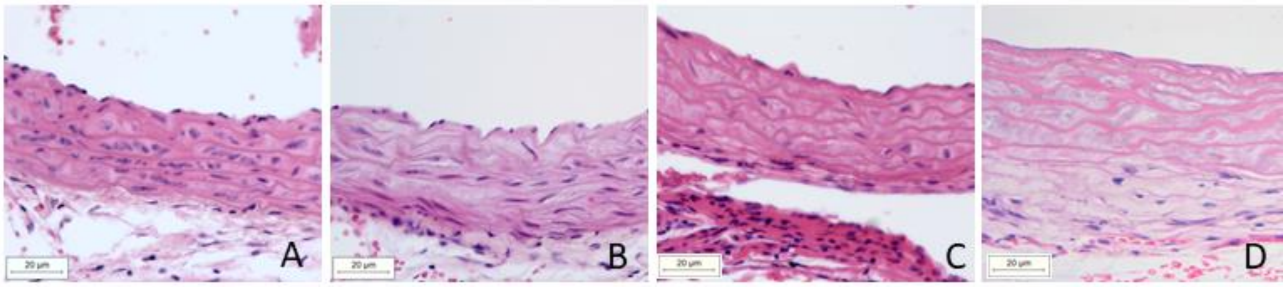

Table S1. Aorta grade of tissue degeneration in tocilizumab-treated *Lmna*$^{G609G/G609G}$ mice. In vehicle-treated animals (NT), 3 out of 6 mice show intermediate to severe aortic lesions (grade 3 or 4). In 3 out of 6 tocilizumab-treated mice (Tocilizumab) the lesion was mild (grade 2).

Aortic arch medium layer alteration grading: increasing severity represented from 1 to 4. (A) Grade1: normal aortic wall. (B) Grade 2: mild decrease of cellularity and myxoid changes (accumulation of acidic mucopolysaccharides) in middle coat (medium layer). Outercoat (adventitia layer) is normal. (C) Grade3: moderate decrease of cellularity and mild myxoid changes in middle coat. (D) Grade 4: severe decrease of cellularity (smooth muscle cells almost absent) and severe myxoid changes in the middle coat extending to the outercoat.

Table S2.

Protein	Forward Primer	Reverse Primer
Human GAPDH	5′TCGGAGTCAACGGATTTGGT 3′	5′TTGCCATGGGTGGAATCATA 3′
Human IL6	5′CCTTCCAAAGATGGCTGAAA 3′	5′ TTTCACCAGGCAAGTCTCCT 3′
Human IL6R	5′CTGGGACTGTGCACTTGCT 3′	5′GGACCCCACTCACAAACAAC 3′
Human sIL6R	5'CAGCTGAGAACGAGGTGTCC 3'	5' TCCACGTCTTCTTGAACCTG 3'
Mouse GAPDH	5' TGTTCCTACCCCCAATGTGT 3'	5' GGTCCTCAGTGTAGCCCAAG 3'
Mouse Progerin	5′GAAGAGCTCCTCCATCACCA 3′	5′ TTCTGGGAGCTCTGGGCT 3′
Mouse Lamin A	5′ GAAGAGCTCCTCCATCACCA 3′	5′TGTGACACTGGAGGCAGAAG 3′
Mouse Lamin A/C	5′ CCACCGAAGTTCACCCTAAA 3′	5′GCCTTCCACACCAAGTCAGT 3′

Table S2. List of primers used in this study.

Supplementary movie.

Movie S1 Motor function is preserved in tocilizumab-treated LmnaG609G/G609G mice. This video shows motor activity of a representative 120 days old maleLmnaG609G/G609G mouse treated with tocilizumab starting at weaning.
